# Effect of spatial scale and latitude on diversity–disease relationships

**DOI:** 10.1002/ecy.2955

**Published:** 2020-01-23

**Authors:** Magnus Magnusson, Ilya R. Fischhoff, Frauke Ecke, Birger Hörnfeldt, Richard S. Ostfeld

**Affiliations:** ^1^ Department of Wildlife, Fish, and Environmental Studies Swedish University of Agricultural Sciences SE‐901 83 Umeå Sweden; ^2^ Cary Institute of Ecosystem Studies Box AB Millbrook New York 12545 USA

**Keywords:** dilution effect, disease, diversity, ecosystem services, infectious diseases, latitude, meta‐analysis, spatial scales

## Abstract

Natural ecosystems provide humans with different types of ecosystem services, often linked to biodiversity. The dilution effect (DE) predicts a negative relationship between biodiversity and risk of infectious diseases of humans, other animals, and plants. We hypothesized that a stronger DE would be observed in studies conducted at smaller spatial scales, where biotic drivers may predominate, compared to studies at larger spatial scales where abiotic drivers may more strongly affect disease patterns. In addition, we hypothesized a stronger DE in studies from temperate regions at mid latitudes than in those from subtropical and tropical regions, due to more diffuse species interactions at low latitudes. To explore these hypotheses, we conducted a meta‐analysis of observational studies of diversity–disease relationships for animals across spatial scales and geographic regions. Negative diversity–disease relationships were significant at small (combined site and local), intermediate (combined landscape and regional), and large (combined continental and global) scales and the effect did not differ depending on size of the study areas. For the geographic region analysis, a strongly negative diversity–disease relationship was found in the temperate region while no effect was found in the subtropical and tropical regions. However, no overall effect of absolute latitude on the strength of the dilution effect was detected. Our results suggest that a negative diversity–disease relationship occurs across scales and latitudes and is especially strong in the temperate region. These findings may help guide future management efforts in lowering disease risk.

## Introduction

The dilution effect (DE) hypothesis predicts that high species diversity frequently reduces pathogen transmission via distinct mechanisms, such as reducing encounter rates between pathogens and susceptible hosts and regulating abundance of susceptible hosts (Keesing et al. 2006). In contrast, the amplification effect hypothesis predicts a positive relationship between diversity and disease if, for example, between‐species transmission is greater than within‐species transmission of a specific pathogen (Keesing et al. 2006). Recent interest in the generality of the dilution effect (DE) is reflected in three quantitative meta‐analyses, viz. Salkeld et al. (2013), Civitello et al. (2015*a*), and Huang et al. (2017). Salkeld et al. (2013) first meta‐analyzed the overall effect of diversity on disease using observational studies, but for a relatively small data set. They found only weak evidence for the generality of the DE across studies. In contrast, Civitello et al. (2015*a*) found overall strong support for the DE, when including a larger sample size than Salkeld et al. (2013) of both experimental and observational studies. Huang et al. (2017) reanalyzed the data from Civitello et al. (2015*a*), comparing DE in plant vs. animal diseases and different types of animal diseases, and concluded that the DE applied generally to diseases of both animals and plants. However, other assessments have come to contrasting conclusions regarding the generality of the dilution effect. According to Cardinale et al. (2012), evidence for the DE acting in plant diseases is strong, but that for an effect of animal diversity on prevalence of animal disease is mixed. Wood et al. (2017) found no general dilution effect across countries in a global scale study.

Negative relationships between diversity and disease arise from specific biotic mechanisms, such as regulation of the abundance of important host species or reduced encounter rates between those hosts and parasites and pathogens (e.g., Keesing et al. 2006). Consequently, one might expect stronger, negative relationships at local and landscape scales where these interactions are thought to manifest themselves. At larger spatial scales, theory predicts, abiotic drivers such as climate may be more important than biotic factors in affecting the distribution and abundance of species (e.g., McGill, 2010). Reduced relative importance of biotic interactions at very large scales could lead to a weak or nonexistent relationship between diversity and disease at these scales. The effect of parasite transmission mode may also vary with spatial scale. For instance, parasites transmitted orally (consumption of contaminated food or water) might be more strongly influenced by climatic factors affecting survival in the environment, whereas directly transmitted parasites might be influenced more strongly by land‐use change affecting infected and susceptible hosts (Loh et al. 2015). A recent study on three emerging pathogens indicated that biotic factors more strongly influenced disease distributions at local than at regional or continental scales (Cohen et al. 2016). Similarly, Ostfeld and Keesing (2017) suggested that so far, the strongest support for the DE derives from relatively small‐scale comparisons in areas suffering diversity loss due to anthropogenic impacts. However, no review so far has investigated the strength of DE at different spatial scales using a systematic, meta‐analytic approach.

In addition to a potential effect of spatial scale on diversity–disease relationships, the geographic location of a community may influence the strength of possible dilution and/or amplification effects. Communities at higher latitudes are typically characterized by lower diversity than are communities at low latitudes (Pianka 1966, Hillebrand 2004). Zoonotic host diversity appears to be highest at low latitudes, while diversity of zoonotic parasites tends to be evenly distributed across latitudes (Han et al. 2016). However, mammalian host species in the species‐poor, subarctic ecosystems harbor a larger number of zoonotic agents as compared with other mammalian hosts elsewhere, and potentially a higher fraction of each population is infected in the subarctic (Han et al. 2016). Also, the insurance hypothesis suggests that ecosystem functioning may be more vulnerable to loss of diversity in species‐poor than in species‐rich ecosystems since high richness of species provide a kind of guarantee that at least some species will maintain functioning even if others fail (Yachi and Loreau 1999, Tilman et al. 2006, Downing et al. 2012). These broad latitudinal patterns could potentially make the diversity–disease relationship stronger in species‐poor regions at high latitudes and weaker in species‐rich regions.

The effect of spatial scale on diversity–disease relationships is relevant to both basic biology and conservation policy. The detection of scale dependence or latitude dependence would help narrow the search for causal mechanisms to those that predominate at a particular spatial scale (Johnson et al. 2015, Cohen et al. 2016). Similarly, if high diversity is associated with reduced disease risk at some scales or latitudes but not others, the management of diversity for health benefits could be directed at relevant scales and latitudes where a desired impact is more likely. In contrast, finding consistent diversity–disease relationships across scales and latitudes, whether positive or negative, would reinforce the generality of those relationships and suggest (although not confirm) consistent underlying causes. To this end, we conducted a review and meta‐analysis of diversity–disease relationships in which we focus on the effect of spatial scale and size of study area on the relationship between diversity and disease, as quantified in observational studies on animal parasites. Our first hypothesis was that the strongest support for the dilution effect will occur at small to medium scales. Second, we hypothesized that the DE acts more strongly at high latitudes than at low latitudes.

## Methods

To conduct a systematic review of applicable published studies, we used combinations of the search terms used by Civitello et al. (2015*a*): parasite, pathogen, diversity, richness, evenness, dilution effect, and decoy effect. We used observational studies and effect sizes from Civitello et al. (2015*a*) and added new literature published after October 2014, which was the last date for their literature search. We conducted our last search on Web of Science in May 2018 (see detailed procedure for the Web of Science search in Appendix S1). For each study, we recorded latitude and spatial scale. We excluded experimental studies because they are often difficult to assign to a specific spatial scale and latitude or rely on a contrived spatial scale. We relied on the original measures in the published studies of both diversity and disease risk, although we recognize that different measures of both key variables exist (Ostfeld et al. 2009). As in Civitello et al. (2015*a*), we used Hedges' *g* as the meta‐analytic measure of effect size of host diversity, typically measured as species richness or evenness, on parasite abundance, which is relevant to risk of transmission, or disease risk. The measure of parasite abundance varied among studies but we strived to use prevalence of parasites within the host populations when possible. That is the measure used in the majority of the reviewed DE‐studies. However, other response variables for testing the diversity–disease relationships were reported, for example human incidence (Derne et al. 2011), disability‐adjusted life years (DALYs; Wood et al. 2017), parasite load (Frank et al. 2016), and number of infected animals (Sintayehu et al. 2017). Johnson et al. (2015) discuss the strengths and limitations of different metrics of disease risk. All equations used to calculate Hedges' *g* from other effect size measures were standard equations obtained from Borenstein et al. (2009), and they are included as supplementary data (Appendix S2; Eqs. S1–S9). In several cases, we obtained raw data by request from authors or manually digitalized published figures to obtain data using the program WebPlotDigitizer 3.9 (Rohatgi 2015). To make the included correlational studies as comparable as possible, when data were available, we fitted linear models to original data sets on diversity–disease relationships to obtain regression coefficients. We also included studies presenting odds ratios.

**Figure 1:**
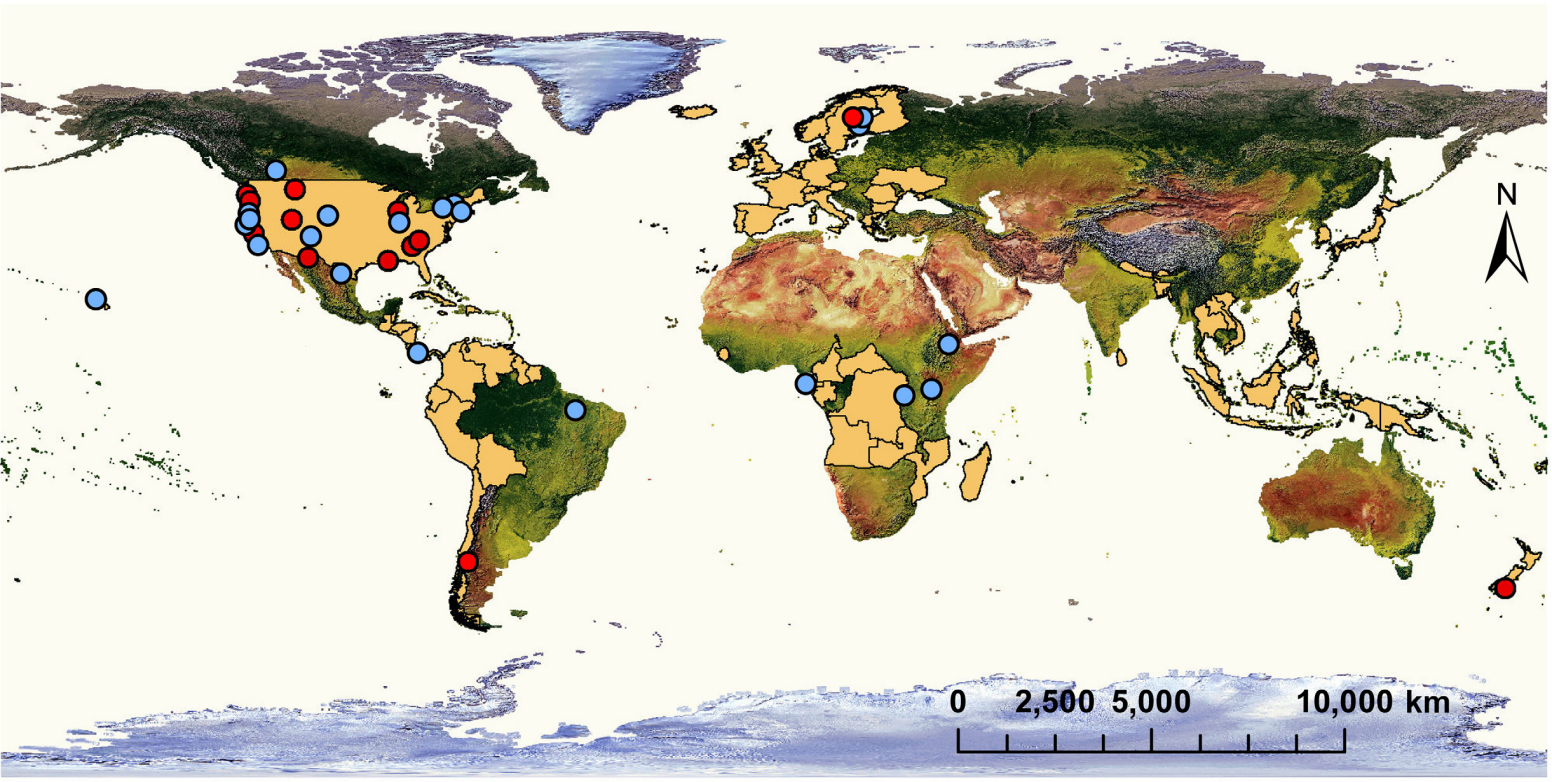
Studies included in the analyses at the (1) small scale, site and local 10 m–10 km (centroid of study areas as red round symbols, *n* = 13 studies, number of effect sizes *k* = 20), (2) intermediate scale, landscape and regional 10–2,000 km (centroid of study areas as blue round symbols, n = 21 studies, *k* = 36), and (3) large scale, continental and global >2,000 km (light brown polygons; *n* = 5 studies, *k* = 27). Note that one study had two effect sizes on two different spatial scales. A kmz file is also available as DataS1 containing study area polygons where overlapping features can be turned on/off.

We initially sorted studies into three different coarse spatial scales (spatial extents) adopted from Pearson and Dawson (2003): (1) site to local, 10 m–10 km; (2) landscape to regional, 10–2,000 km; and (3) continental to global, >2,000 km. These scales reflected the area over which diversity and the relevant disease metric were estimated. We then used the original six spatial scales described in Pearson and Dawson (2003) for a follow‐up, narrow‐scale analysis: (1) site, 10–1,000 m; (2) local, 1–10 km; (3) landscape, 10–200 km; (4) regional, 200–2,000 km; (5) continental, 2,000–10,000 km; and (6) global, >10,000 km. We used linear distances (m) instead of area measures to standardize assignment of spatial scales. Some studies used linear transects spanning a certain distance covering a diversity gradient, while others used area measures. When the original study design reported an area (rather than linear distance), we used the longest diagonal in a GIS‐polygon covering the study area to estimate the appropriate scale in meters. We also conducted a separate analysis where size of each study area polygon (ha) was treated as a continuous variable. All study areas at landscape, regional, continental, and global scales were digitized on a global map as polygons, while site and local scales were marked as point features. The resulting global map is available as a kmz file with information about polygon size in hectares for each study area (Data S1) and in a simplified version in Fig. 1.

For the geographic region analysis, we sorted the studies of diversity–disease relationships into the three geographic regions: (1) tropical (<23.5°); (2) subtropical (23.5°–35°), and (3) temperate regions (>35°–66.5°). No studies providing effect sizes were found in the polar region (>66.5°). If a study area polygon stretched over two regions, we assigned these studies to the region where the centroid of the study area polygon was placed. As a complementary analysis, we also used absolute latitude of the centroid as a continuous variable. However, we omitted large‐scale studies (*n* = 2) at the continental‐global scale that included several countries spanning all three geographic regions, as these studies could not be properly classified as belonging to one of the regions or a specific latitude.

### Statistical analyses

Six separate mixed‐effects meta‐analyses were performed: (1) estimation of the overall effect size for the whole data set, (2) an analysis at the three coarser spatial scales, (3) an analysis at the six narrower spatial scales, (4) an analysis using study area in hectares as a continuous variable, (5) an analysis for the three geographic regions, and (6) an analysis treating absolute latitude as a continuous variable. For these six statistical models, we used the rma.mv function in the metafor package in R allowing the specification of random effects structures to each model (Viechtbauer 2010, 2016). Pair‐wise comparisons between the different spatial scales in the derived mixed‐effects models and a comparison between the three geographic regions were also made using the metafor package. To detect possible publication bias we used a funnel plot followed by Eggers regression test to estimate funnel plot asymmetry (Egger et al. 1997). A significant correlation between effect size and variance in effect size can arise due to publication bias, dependencies between the sampling variance and the effect size, or due to other sources of heterogeneity in study subjects and methods (Lau et al. 2006, Koricheva et al. 2013, Civitello et al. 2015*a*, 2015*b*). For each of the six different models, the amount of heterogeneity was estimated using restricted maximum likelihood, and separate random effects were assigned to study number and parasite species to account for underlying heterogeneity in concordance with Civitello et al. (2015*a*).

## Results

Our literature search on observational field‐based studies on animal parasites revealed 83 effect sizes from 38 studies spanning 42 different parasite types (22 distinct species and 20 families or species groups; see Data S2). The range of species richness used in the diversity measures in our reviewed studies ranged from 1 to a global data set composed of richness data from Jenkins et al. (2013) covering >21,000 birds, mammals, and amphibians. The global data set was used in Wood et al. (2017) in their study of diseases in 60 countries in which they constructed a diversity measure of species richness per unit area. Twenty‐nine effect sizes were derived from field observational studies in the Civitello et al. (2015*a*) database while 54 effect sizes were obtained from our search of more recent publications (Data S2). We omitted five effect sizes from the Civitello et al. (2015*a*) database since they did not meet our selection criteria (Appendix S1).

The total number of effect sizes (*k*) per coarse spatial scale were (1) site and local, *k* = 20 from 13 studies; (2) landscape and regional, *k* = 36 from 21 studies; (3) continental and global, *k* = 27 from five studies. At the narrower scales, the number of effect sizes per spatial scale were (1) site, *k* = 6 from four studies; (2) local, *k* = 14 from nine studies; (3) landscape, *k* = 18 from 12 studies; (4) regional, *k* = 18 from 12 studies; (5) continental, *k* = 5 from four studies; (6) global, *k* = 22 from one study. One study had two effect sizes at two different spatial scales.

For the comparison of different geographic regions, only 60 effect sizes from 36 studies were derived since two of the large‐scale studies in the overall data set (containing 23 effect sizes) covered all three geographic regions and were omitted. In the tropical region, we found *k* = 17 from seven studies, the subtropical region, *k* = 7 from five studies and the temperate region, *k* = 36 from 24 studies.

Overall, we found a significant negative effect of diversity on disease (*g* = −0.54 ± 0.13 [mean ± SE], *P* < 0.0001; Appendix S3), consistent with a general dilution effect across studies and also with the findings of Civitello et al. (2015*a*). The Egger's regression test for funnel plot asymmetry was significant using variance of Hedges' *g* as moderator to the mixed‐effects model (*P* < 0.0001). This significant asymmetry in the funnel plot analyses indicates that there may have been a bias toward the publication of studies with significant positive or negative relationships between diversity and disease and against the publication of studies with no significant relationships. To detect influential outliers, we examined the funnel plot of the overall model and identified two effect sizes from two separate studies as outliers (Appendix S3). By excluding these effect sizes in two separate leave‐one‐out analyses we evaluated the sensitivity of our analysis to statistical outliers. We found that both leave‐one‐out models showed highly significant, negative associations between diversity and disease (*g* = −0.69 ± 0.19, *P* < 0.001 and *g* = −0.81 ± 0.24, *P* < 0.001, respectively), indicating that the perceived outliers had no detectable effect on conclusions.

**Figure 2:**
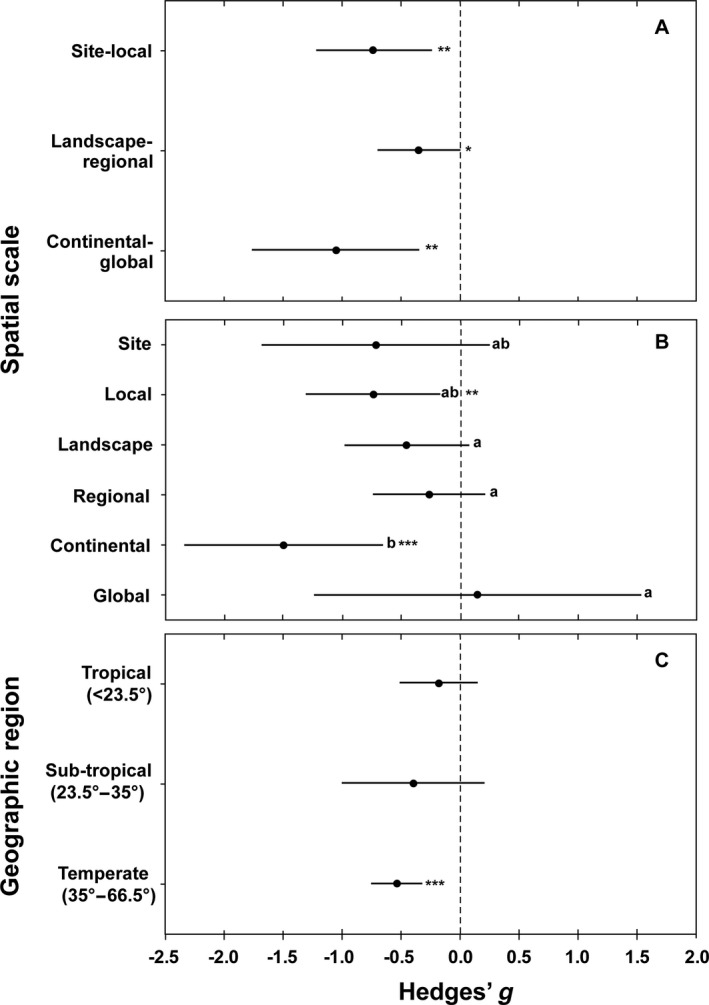
(A) Mean effect sizes (and 95% confidence intervals; Hedges' *g*) of a mixed‐effect meta‐analysis of the dilution effect in 38 field observational studies on parasites for three coarser spatial scales using 83 effect sizes (*k*) in total: (1) site and local 10 m–10 km (*k* = 20), (2) landscape and regional 10–2,000 km (*k* = 36), and (3) continental and global 2,000–>10,000 km (*k* = 27) scales. Note that one study had two effect sizes on two different spatial scales. The mean effect of biodiversity on parasite abundance did not differ between scales. (B) Mean effect sizes (and 95% confidence intervals; Hedges' *g*) of a mixed‐effect meta‐analysis of the dilution effect in 38 field observational studies on parasites for six narrow spatial scales using 83 effect sizes: (1) site 10–1,000 m (*k* = 6), (2) local 1–10 km (*k* = 14), (3) landscape 10–200 km (*k* = 18), (4) regional 200–2,000 km (*k* = 18), (5) continental 2,000–10,000 km (*k* = 5), and (6) global >10,000 km (*k* = 22) scales. Different lowercase letters indicate significant differences (*P* ≤ 0.05) in the mean effect of biodiversity on parasite abundance among different spatial scales. The mean effect of biodiversity on parasite abundance differed significantly (*P* ≤ 0.05) between the continental scale vs. landscape, regional, and global scale. (C) Mean effect sizes (and 95% confidence intervals; Hedges' *g*) of a mixed‐effect meta‐analysis of the dilution effect in 36 field observational studies with 60 effect sizes (*k*) on parasites in the tropical (<23.5°; *k* = 17), subtropical (23.5°–35°; *k* = 7) and temperate region (>35°–66.5°; *k* = 36). The mean effect of biodiversity on parasite abundance did not differ between the three subgroups. The 0 line delimits dilution (negative values) from amplification (positive values) effects of diversity on disease risk. Asterisks indicate significant (*P ≤ 0.05; **P ≤ 0.01; ***P ≤ 0.001) differences from zero.

For the meta‐analysis using the three, broad categories of scale, we found a significant negative effect of diversity on disease at the site‐local (*g* = −0.73 ± 0.25, *P* < 0.01), landscape‐regional (*g* = −0.35 ± 0.18, *P* = 0.05) and continental‐global scale (*g* = −1.05 ± 0.36, *P* < 0.01, Fig. 2A, Appendix S3). The negative effect of diversity on disease did not differ among the scales (Fig. 2A).

In the analysis using six narrow spatial scales we found a significant negative effect of diversity on disease at the local (*g* = −0.74 ± 0.29, *P* = 0.01) and continental scale (*g* = −1.50 ± 0.43, *P* < 0.001), but not at the site (*g* = −0.72 ± 0.49, *P* = 0.14), landscape (*g* = −0.46 ± 0.27, *P* = 0.09), regional (*g* = −0.26 ± 0.24, *P* = 0.27) or global (*g* = 0.14 ± 0.71, *P* = 0.84) scales (Fig. 2B; Appendix S3). The dilution effect was stronger at the continental scale compared with the landscape, regional, and global scales. The other scales did not differ from one another (Fig. 2B). In the analysis using area in hectares as a continuous variable (log‐transformed) we found no significant effect of area on the strength of the dilution effect (estimate = 0.01 ± 0.04, *P* = 0.87; Appendix S4).

For the geographic region meta‐analysis, studies performed in the temperate region showed a highly significant dilution effect (*g* = −0.54 ± 0.11, *P* < 0.0001) while the diversity–disease relationship was nonsignificant in the tropical (*g* = −0.18 ± 0.17, *P* = 0.27) and subtropical (*g* = −0.39 ± 0.31, *P* = 0.20) regions (Fig. 2C; Appendix S3). There were no significant differences in any paired geographic comparisons. In the analysis using absolute values of the latitude (centroid of each study area polygon) as a continuous variable, we found no significant effect of latitude on the strength of the dilution effect (estimate = −0.01 ± 0.00, *P* = 0.11; Appendix S4).

## Discussion

According to our meta‐analysis considering several orders of magnitude in the spatial scale at which diversity and disease were measured in published studies (Fig. 2A), the relationship between diversity and disease risk is negative at site‐local, landscape‐regional, and continental‐global scale. Also, no effect of area as a continuous variable on the strength of the dilution effect could be detected (Appendix S4). This is inconsistent with our initial hypothesis that the dilution effect is strongest at smaller and weaker at larger scales. However, the continental and global scales were significantly different from each other when analyzed in a narrower scale domain (Fig. 2B) with continental scale being highly significantly negative and the global scale being positive but nonsignificant. This indicates potentially contrasting results on the diversity–disease relationship between different large‐scale realms.

The design of large‐scale studies at continental and global scales often includes data on disease incidence in humans as the response variable in the diversity–disease relationship (see, e.g., Derne et al. 2011, Hofmeester 2016) or, as in Wood et al. (2017), disability‐adjusted life years (DALYs, a measure of disease burden used to facilitate comparison of diseases with lethal and sublethal health effects [Murray et al. 2012]). Such approaches and the direction of the estimates from the resulting correlations should be evaluated with caution. Disease risk is a function of both environmental factors such as diversity loss affecting parasite–host interactions, and socioeconomic factors such as infrastructure affecting human exposure to parasites at any given level of risk (Ostfeld et al. 2009). Ecological studies tend to focus on the abundance or transmission probabilities of parasites, whereas some epidemiological studies include socioeconomic factors or fail to distinguish between the two. These observations illustrate the broader issue that differing definitions of disease risk can result in different conclusions, particularly if definitions vary with scale of the analysis. The need for improving analyses of linkages between measures of risk of exposure and actual disease incidence has been discussed by Salkeld et al. (2013), Johnson et al. (2015), and others. To better account for this, we propose a two‐step approach for future studies: (1) estimating the relationship between diversity and disease risk as measured by, for example, the abundance of parasites, infected reservoir hosts, or infected vectors, and (2) analyzing the relationship between disease risk and human disease outcomes. Such an approach may reveal whether high disease risk caused by environmental changes is indeed correlated with human incidence as assumed by the previously mentioned large‐scale studies. If not, relationships between socioeconomic or human behavior factors and disease patterns may need to be analyzed separately from the ecological relationships.

Our analysis using the latitude of each study area as a continuous, independent variable suggests that latitude does not modulate the strength of diversity–disease relationships. However, when we analyzed diversity–disease relationships after aggregating studies into latitudinal categories, we found that studies from the temperate zone showed the strongest dilution effects, while those from tropical and subtropical regions showed nonsignificant relationships. This partially supports our second hypothesis. However, we note that the ability to assess effects of geographic zone is limited by the absence of studies from the polar region (>66.5°) and the preponderance of studies from temperate North America. It remains plausible that high diversity communities in the tropics tend to have more diffuse species interactions, due to nonrandom structuring of ecological communities, which might lead to a weaker relationship between diversity and disease (Johnson et al. 2013).

Our approach to study scale dependency of the DE, using a meta‐analytic framework and calculating common effect sizes for each study to standardize parasite abundance, represents the recommended method of conducting meta‐analyses (Borenstein et al. 2009, Koricheva et al. 2013). Such approaches have been used in all previous meta‐analyses on the generality of the DE (Salkeld et al. 2013, Civitello et al. 2015*a*, Huang et al. 2017), allowing investigators to combine studies reporting different measures of both diversity and disease risk into one analysis. A disadvantage is that other properties of the diversity–disease relationship, such as its shape, cannot be studied in detail. For example, Halliday and Rohr (2018) recently used regression models fitted to a large number of studies to study the shape of diversity–disease regression curves instead of standardizing parasite abundance as we have done. They report, based on skewness of regression curves, a scale dependency for DE at local scales and also some positive diversity–disease correlations at larger scales. This latter finding contrasts with our finding of a strongly negative diversity–disease relationship at the largest scale as well. We did not detect significant amplification effects (sensu Keesing et al. 2006) at any spatial scale. It is possible that different measures of disease risk used by Halliday and Rohr (2018) and the present study may at least in part account for these contrasting results. Our detection of funnel plot asymmetry suggests that studies with nonsignificant findings may be less likely to be published. Funnel plot asymmetry, however, can also be caused by other factors, such as differences in study populations (e.g., taxa of hosts or parasites) or methods (e.g., metrics of disease; Borenstein et al. 2009, Koricheva et al. 2013). Also, when complex dependencies between effect sizes arise, as may be the case for the present data set, funnel plots may be misleading and not appropriate (Lau et al. 2006). Because they emphasize the standard deviation of the individual studies included in meta‐analyses, Hedges' *g* and Cohen's *d* statistics have been criticized for use with biological data (Osenberg et al. 1997). We chose to use Hedges' *g* as a measure because it allows us to combine effect sizes reported from different study types (Borenstein et al. 2009) and has been applied previously in diversity–disease meta‐analyses. For data sets with small sample sizes, such as ours, and with unequal sampling variance in studies using paired groups, Hedges' *g* is also the suggested measure (Koricheva et al. 2013).

Our study addresses spatial scale and latitude as potential causes of variation in the nature and strength of diversity–disease relationships. Of course, many other factors can potentially affect these relationships. For example, Civitello et al. (2015*a*) and Huang et al. (2017) tested whether mode of transmission, type of parasite (e.g., macro‐ vs. micro‐; simple vs. complex life cycle), or type of host (e.g., human or non‐human) affected diversity–disease relationships. They found consistent, negative diversity–disease relationships across systems irrespective of transmission mode, parasite type, and host range, host type, and study design (observational vs. manipulative). Other sources of variation, such as ecosystem type (e.g., terrestrial vs. aquatic), disturbance regime, and parasite virulence, remain to be assessed. By now addressing the impacts of spatial scale and latitude, however, we have broadened the overall assessment of how generally the DE is observed in nature.

To conclude, our study indicates that negative relationships between diversity and disease are consistent across spatial scales. Absolute latitude does not modulate the strength of the diversity–disease relationship. However, the negative relationship is significant and strong in studies conducted in temperate‐zone locations but weak or absent in subtropical and tropical studies. Our results can help generate hypotheses about the causes of patterns, but they cannot by themselves thoroughly evaluate those hypotheses. We hope that experimental and rigorous comparative studies will be designed to better infer causality. The meta‐analytic results suggest that conservation actions with a focus on combating the loss of native biodiversity may be effective in reducing disease risk at any scale. Although conservation actions at local scales are often most feasible, policies concerning biodiversity conservation can address any scale from the local to the national and international. However, information on the specific mechanisms that govern diversity–disease relationships in any specific location, and for any specific disease, will undoubtedly help clarify if and when diversity conservation is likely to also reduce infectious disease. Whether and how these results, linking high diversity to reduced disease risk at multiple scales and a wide range of latitudes, can be used to promote and implement joint environmental and health policy goals should be the subject of future research efforts.
