# Supplementary material for: Effect of spatial scale and latitude on diversity–disease relationships
Source: Ecology. 2020 Jan 23;101(3):e02955. doi: 10.1002/ecy.2955 (PMC7078972; doi:10.1002/ecy.2955)
Supplement: Supplementary file 1 [file ECY-101-e02955-s001.pdf]

Supporting Information. Magnus Magnusson, Ilya Fischhoff, Frauke Ecke, Birger Hörnfeldt, Richard S. Ostfeld. 2020. Effect of spatial scale and latitude on diversity–disease relationships. *Ecology.*

Appendix S1

Search strategy for identifying articles on the dilution effect at different spatial scales

Criteria:

- Include all observational studies published prior to October 2014 in Civitello et al. (2015) fulfilling the criteria specified below.
- Search the Web of Science for articles published in October 2014 – May 2018. Civitello et al. (2015) made their last search in October 2014.
- Keywords the same as in Civitello et al. (2015): Dilution effect, decoy effect, parasite, pathogen, diversity, richness, evenness.
- Include only observational studies.
- Exclude studies on herbivores.
- Language: English.
- Publication type: Peer-reviewed articles, book chapters, data papers or proceedings paper.
- Two co-authors refined articles by title and abstract from the initial search independently to avoid selection bias.

Studies from Civitello et al. (2015):

Twenty-nine effect sizes from 19 observational studies were retrieved from the Civitello et al. (2015) database. Five effect sizes that Civitello et al. (2015) included were omitted since they did not fit our selection criterias specified above:

- Two effect sizes from Salkeld et al. (2013) – based on unpublished data with none or unclear description of study areas.
- One effect size from Lau et al. (2005) - herbivore study.
- Two effect sizes from Soria et al. (1995) - in Spanish.

Advanced search in Web of Science:

No 1. TS = ((“dilution effect” OR “decoy effect”) AND ("richness" OR "pathogen" OR "evenness" OR "parasite" OR "diversity"))

- ***Retrieved results: 180 (15/5 2018)***

No 2. TS = ("dilution effect" OR "decoy effect")

- ***Retrieved results: 1073 (15/5 2018)***

No 2 Refined by: TOPIC: (richness)
Retrieved results: ***49***
No 2 Refined by: TOPIC: (pathogen)
Retrieved results: ***135***
No 2 Refined by: TOPIC: (evenness)
Retrieved results: ***6***
No 2 Refined by: TOPIC (parasite)

Retrieved results: ***118***
No 2 Refined by: TOPIC (diversity)
Retrieved results: ***116***

- ***Refined by title to: 96***
- ***Refined by abstract to: 46***
- ***Refined by full text to: 19 articles (54 effect sizes)***

LITERATURE CITED

Civitello, J. D., J. Cohen, H. Fatima, N. T. Halstead, J. Liriano, T. A. McMahon, C. Nicole Ortega, E. L. Sauer, T. Sehgal, S. Young and J. R. Rohr. 2015. Biodiversity inhibits parasites: Broad evidence for the dilution effect. Proceedings of the National Academy of Sciences, USA 112:8667-8671.

Lau, J. A. and S. Y. Strauss. 2005. Insect herbivores drive important indirect effects of exotic plants on native communities. Ecology 86: 2990-2997.

Salkeld, D. J., K. A. Padgett and J. H. Jones. 2013. A meta-analysis suggesting that the relationship between biodiversity and risk of zoonotic pathogen transmission is idiosyncratic. Ecology Letters 16: 679-686.

Soria, F. J., M. Villagran, R. Del Tio and M. E. Ocete. 1995. Incidencia de Curculio elephas Gyll. (Col., Curculionidae) en alcornocales y encinares del parque natural Sierra Norte de Sevilla. Boletin de Sanidad Vegetal Plagas 21: 195-201.
