# Supplementary material for: Effect of spatial scale and latitude on diversity–disease relationships
Source: Ecology. 2020 Jan 23;101(3):e02955. doi: 10.1002/ecy.2955 (PMC7078972; doi:10.1002/ecy.2955)
Supplement: Supplementary file 2 [file ECY-101-e02955-s002.pdf]

Supporting Information. Magnus Magnusson, Ilya Fischhoff, Frauke Ecke, Birger Hörnfeldt, Richard S. Ostfeld. 2020. Effect of spatial scale and latitude on diversity–disease relationships. *Ecology.*

Appendix S2

Eq.S1-Eq.S9

Standard equations used to calculate Hedges'g from correlational studies (all equations are retrieved from Borenstein et al. (2009)).

Eq.S1

To transform correlation coefficient r to Fischer's Z

$Z = 0.5 \times \ln((1 + r) / (1 - r))$

Eq.S2

To retrieve variance of r (Vr)

$Vr = ((1 - r^2)^2) / (n - 1)$

Eq.S3

To retrieve variance of Fischer's Z (Vz)

$Vz = 1 / (n - 3)$

Eq.S4

To retrieve standard error of Fischer's Z (SEz)

$SEz = \sqrt{Vz}$

Eq.S5

To retrieve Cohen's d

$d = 2r / (\sqrt{(1 - r^2)})$

Eq.S6

To retrieve Variance of d (Vd)

$Vd = 4Vr / (1 - r^2)^3$

Eq.S7

To retrieve correction factor J

$J = 1 - (3 / (4df - 1))$

Eq.S8

To retrieve Hedges' g from Cohen's d

$g = J \times d$

Eq.S9

To retrieve variance of g (Vg) from variance of d (Vd)

$Vg = J^2 \times Vd$

LITERATURE CITED

Borenstein, M., L. V. Hedges, J. P. T. Higgins and H. R. Rothstein. 2009. Introduction to Meta-Analysis. John Wiley & Sons Ltd. New Jersey, USA.
