# Supplementary material for: Effect of spatial scale and latitude on diversity–disease relationships
Source: Ecology. 2020 Jan 23;101(3):e02955. doi: 10.1002/ecy.2955 (PMC7078972; doi:10.1002/ecy.2955)
Supplement: Supplementary file 3 [file ECY-101-e02955-s003.pdf]

Supporting Information. Magnus Magnusson, Ilya Fischhoff, Frauke Ecke, Birger Hörnfeldt, Richard S. Ostfeld. 2020. Effect of spatial scale and latitude on diversity–disease relationships. *Ecology*.

Appendix S3

Fig. S1. Funnel plot for estimating publication bias in the overall model (k = 83 from 38 studies). Two outliers are shown in red (Mills et al. 2005; Rendon-Franco et al. 2014). Various levels of statistical significance of the points/studies are indicated by the shaded regions; white: $P > 0.10$, light grey: P = 0.05-0.10, dark grey: P = 0.01-0.05, area outside the funnel shape: $P < 0.01$.

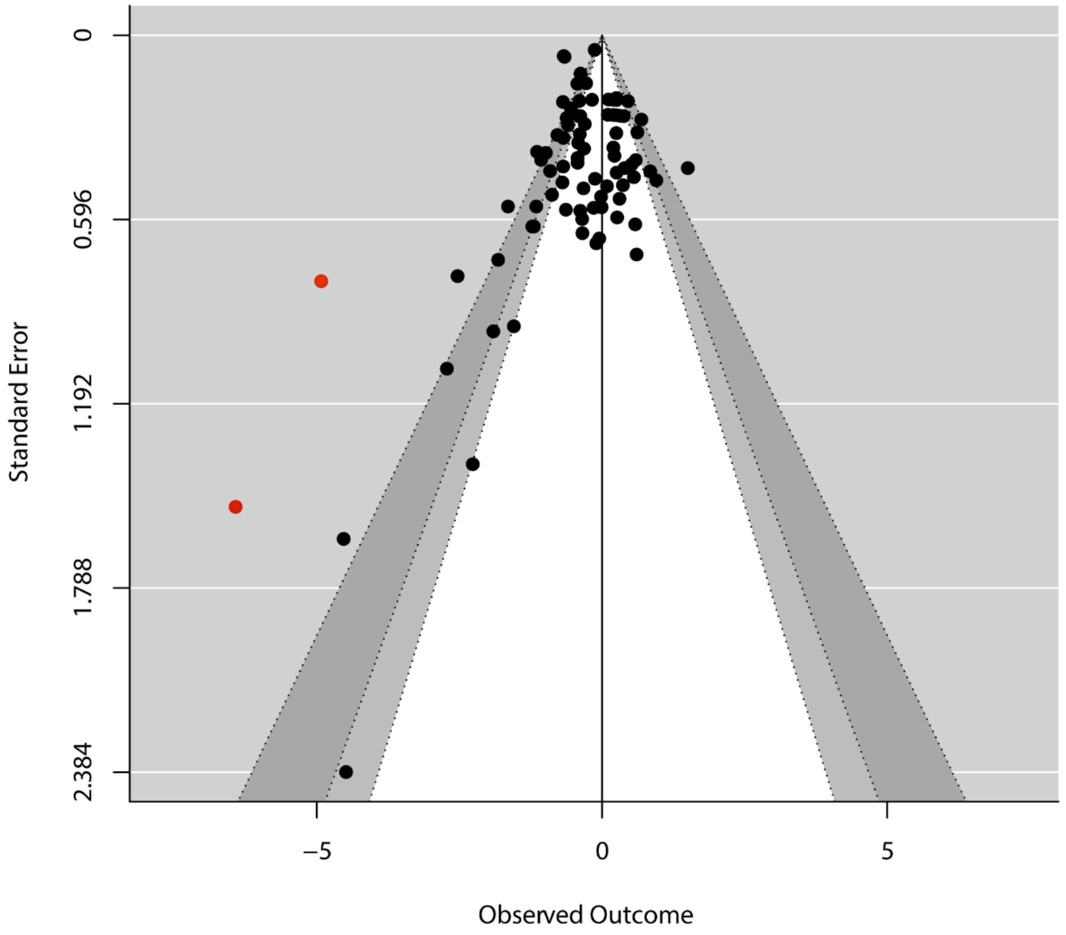

Table S1. Model summaries for the (a) overall, (b) coarse spatial scale, (c) narrow spatial scale and (d) geographic region model. Significant values in bold.

(a)

Estimate	SE*	Z-value	*P*-value	Lower CI**	Upper CI
-0.54	0.13	-4.22	**<0.0001**	-0.79	-0.29

(b)

Spatial scale	Estimate	SE	Z-value	*P*-value	Lower CI	Upper CI
1. Site – local	-0.73	0.25	-2.94	**<0.01**	-1.22	-0.24
2. Landscape – regional	-0.35	0.18	-1.94	**0.05**	-0.70	0.00
3.Continental – global	-1.05	0.36	-2.88	**<0.01**	-1.76	-0.34

(c)

Spatial scale	Estimate	SE	Z-value	*P*-value	Lower CI	Upper CI
1. Site	-0.72	0.49	-1.46	0.14	-1.68	0.25
2. Local	-0.74	0.29	-2.56	**0.01**	-1.31	-0.17
3. Landscape	-0.46	0.27	-1.69	0.09	-0.99	0.07
4. Regional	-0.26	0.24	-1.10	0.27	-0.74	0.21
5. Continental	-1.50	0.43	-3.49	**<0.001**	-2.34	-0.66
6. Global	0.14	0.71	0.20	0.84	-1.24	1.52

(d)

Region	Estimate	SE	Z-value	*P*-value	Lower CI	Upper CI
1. Tropical	-0.18	0.17	-1.10	0.27	-0.51	0.14
2. Sub-tropical	-0.39	0.31	-1.28	0.20	-1.00	0.21
3. Temperate	-0.54	0.11	-4.83	**<0.0001**	-0.76	-0.32

* SE = Standard error

** CI = Confidence interval
