# Supplementary material for: Effect of spatial scale and latitude on diversity–disease relationships
Source: Ecology. 2020 Jan 23;101(3):e02955. doi: 10.1002/ecy.2955 (PMC7078972; doi:10.1002/ecy.2955)
Supplement: Supplementary file 4 [file ECY-101-e02955-s004.pdf]

Supporting Information. Magnus Magnusson, Ilya Fischhoff, Frauke Ecke, Birger Hörnfeldt, Richard S. Ostfeld. 2020. Effect of spatial scale and latitude on diversity–disease relationships. *Ecology*.

Appendix S4

Table S1. Model summaries for (a) the effect of area (ha; log-transformed) and (b) absolute values of latitude (centroid of study area polygons) on the strength of the dilution effect.

(a)

Variable	Estimate	SE*	Z-value	*P*-value	Lower CI**	Upper CI
Intercept	-0.59	0.28	-2.09	**0.04**	-1.14	-0.04
log area	0.01	0.04	0.16	0.87	-0.09	0.10

(b)

Variable	Estimate	SE	Z-value	*P*-value	Lower CI	Upper CI
Intercept	-0.17	0.18	-0.95	0.34	-0.53	0.18
abs latitude	-0.01	0.00	-1.59	0.11	-0.02	0.00

* SE = Standard error
** CI = Confidence interval
