# Supplementary material for: Effect of spatial scale and latitude on diversity–disease relationships
Source: Ecology. 2020 Jan 23;101(3):e02955. doi: 10.1002/ecy.2955 (PMC7078972; doi:10.1002/ecy.2955)
Supplement: Supplementary file 5 [file ECY-101-e02955-s005.pdf]

Magnusson, M., I. R. Fischhoff, F. Ecke, B Hörnfeldt, and R. S. Ostfeld. 2020. Effect of spatial scale and latitude on diversity–disease relationships. *Ecology.*

Data S1

Study map with polygons and point features for all studies included in the meta-analyses (n = 38)

Authors

Magnus Magnusson
Department of Wildlife, Fish and Environmental Studies, Swedish University of Agricultural Sciences
SE-901 83 Umeå, Sweden
magnus.magnusson@slu.se

Ilya R. Fischhoff
Cary Institute of Ecosystem Studies
Box AB, Millbrook, New York 12545 USA
fischhoffi@caryinstitute.org

Frauke Ecke
Department of Wildlife, Fish and Environmental Studies, Swedish University of Agricultural Sciences
SE-901 83 Umeå, Sweden
frauke.ecke@slu.se

Birger Hörnfeldt
Department of Wildlife, Fish and Environmental Studies, Swedish University of Agricultural Sciences
SE-901 83 Umeå, Sweden
Birger.Hornfeldt@slu.se

Richard S. Ostfeld
Cary Institute of Ecosystem Studies
Box AB, Millbrook, New York 12545 USA
ostfeldr@caryinstitute.org

File list (file found within DataS1.zip)

`DataS1.kmz`

Description

`DataS1.kmz`– File containing polygons and point features for all study areas (n = 38) used in the meta-analyses where overlapping study area polygons can be turned on/off. For each study, information about the area in hectares is given. The file can be opened using Google Earth Pro.
