# Supplementary material for: Effect of spatial scale and latitude on diversity–disease relationships
Source: Ecology. 2020 Jan 23;101(3):e02955. doi: 10.1002/ecy.2955 (PMC7078972; doi:10.1002/ecy.2955)
Supplement: Supplementary file 6 [file ECY-101-e02955-s006.pdf]

Magnusson, M., I. R. Fischhoff, F. Ecke, B Hörnfeldt, and R. S. Ostfeld. 2020. Effect of spatial scale and latitude on diversity–disease relationships. *Ecology.*

Data S2

Database of all studies (n = 38) used in the meta-analyses

Authors

Magnus Magnusson
Department of Wildlife, Fish and Environmental Studies, Swedish University of Agricultural Sciences
SE-901 83 Umeå, Sweden
magnus.magnusson@slu.se

Ilya R. Fischhoff
Cary Institute of Ecosystem Studies
Box AB, Millbrook, New York 12545 USA
fischhoffi@caryinstitute.org

Frauke Ecke
Department of Wildlife, Fish and Environmental Studies, Swedish University of Agricultural Sciences
SE-901 83 Umeå, Sweden
frauke.ecke@slu.se

Birger Hörnfeldt
Department of Wildlife, Fish and Environmental Studies, Swedish University of Agricultural Sciences
SE-901 83 Umeå, Sweden
Birger.Hornfeldt@slu.se

Richard S. Ostfeld
Cary Institute of Ecosystem Studies
Box AB, Millbrook, New York 12545 USA
ostfeldr@caryinstitute.org

File list (file found within DataS2.zip)

DataS2.csv

Description

DataS2.csv – Database containing all information about the studies used in the meta-analyses. Below is the legend to all columns.

Effect size = All effect sizes (n = 83)
Study no = All included studies (n = 38)
Name = Publication name
g = Hedges' g effect size measure
Variance g = Variance of hedges' g
Size (ha) = Size in hectares of each study area
Narrow scale = (1) site 10-1000 m, (2) local 1-10 km, (3) landscape 10-200 km, (4) regional 200-2000 km, (5) continental 2000-10 000 km, and (6) global > 10 000 km scale
Coarse scale = (1) site and local 10 m-10 km, (2) landscape and regional 10 - 2000 km, and (3) continental and global 2000 - > 10 000 km scale
Bioregion = (1) Tropic zone <23.5° latitude, (2) Sub-tropic zone 23.5 - 35° latitude, (3) Temperate zone >35° latitude
Abs_latitude = Absolute latitude of the centroid in each study area polygon
Year = Study year
Country = Country, countries or region where the study was conducted
Location = Detailed description of location
Data origin = Information showing if the effect size was included in Civitello et al. (2015) or if is a new effect size in the current study
Parasite = Parasite species, family or parasite group
Disease / parasite cause = Name of disease caused by the parasite or other type of parasite causes
Sample size (corr.studies) = Sample sizes for the correlational studies (excluding effect sizes from Civitello et al. 2015
n1(group studies) = Sample sizes for group 1 in studies comparing groups (excluding effect sizes from Civitello et al. 2015)
n2(group studies) = Sample sizes for group 2 in studies comparing groups (excluding effect sizes from Civitello et al. 2015)
Taxa = Species taxa/group in focus for the effect size (excluding effect sizes from Civitello et al. 2015)
Predictor = Predictor variable for the diversity – disease correlation. Only available for the correlational studies (excluding effect sizes from Civitello et al. 2015)
Response = Response variable for the diversity – disease correlation. Only available for the correlational studies (excluding effect sizes from Civitello et al. 2015)
Human parasite = Yes/No – information on whether it is a human parasite or not.
